# Preoperative conjunctival flora and antibiotic susceptibility in cataract surgery patients at Ibn Al-Haitham teaching eye hospital in Baghdad, Iraq

**DOI:** 10.1186/s12348-025-00471-z

**Published:** 2025-02-27

**Authors:** Mohammed A. Fenjan, Furkaan Majied Hamied, Ahmed Sermed Al Sakini, Hamzeh Khorsheed, Sandra Thair Al-aish

**Affiliations:** 1 Department of ophthalmology, Ibn Al Haitham Teaching Eye Hospital, Baghdad, Iraq; 2 Department of ophthalmology, College of Medicine, Al-Qadisya University, Qadisya, Syria; 3 Department of surgery, College of Medicine, University of Baghdad, Baghdad, Iraq; 4 Jordanian University of Science and Technology, Irbid, Jordan

**Keywords:** Conjunctival flora, Antimicrobial susceptibility, Cataract surgery, Iraq

## Abstract

**Background:**

Endophthalmitis is a rare but severe eye inflammatory disorder caused by bacterial infection that can occur after cataract surgery. Most bacteria are part of the patient’s natural flora, and even with antibiotic treatment, it causes considerable ocular morbidity and vision loss.

**Purpose:**

To investigate the preoperative conjunctival flora and their antibiotic susceptibility in patients undergoing cataract surgery at Ibn Al-Haitham Teaching Eye Hospital, a tertiary ophthalmology center in Baghdad, Iraq.

**Methods:**

This cross-sectional, single-center study based on conjunctival swabs of patients prior to cataract surgery and cultured using blood agar, chocolate agar, MacConkey agar, and Sabouraud agar. Bacterial isolates were identified using Gram staining and biochemical tests, and antibiotic sensitivity was determined using the disc diffusion method according to CLSI guidelines. Statistical analysis was done by SPSS Statistics Version 23.

**Results:**

A total of 200 patients (200 conjunctival swabs of consecutive 200 eyes) scheduled for cataract surgery were included. Positive cultures were found in 45 (24%) patients. Coagulase-negative *Staphylococcus epidermidis* was the most frequently isolated microorganism (75% of isolates), followed by *Staphylococcus aureus* (14.58%), *Aspergillus* species (6.25%), and *E. coli* (4.17%). *S. epidermidis* showed the highest sensitivity to ofloxacin (97.2%) and chloramphenicol (94.4%) and the lowest sensitivity to fusidic acid (11.1%) and ceftazidime (5.6%). *S. aureus* exhibited the highest sensitivity to chloramphenicol (100%) and tobramycin (85.7%) but was completely resistant to ceftazidime and fusidic acid (100% resistance). *E. coli* isolates were 100% sensitive to ciprofloxacin, ofloxacin, and chloramphenicol.

**Conclusion:**

The study revealed conjunctival microbial colonization in 24% of cataract surgery candidates, with S. epidermidis being the most prevalent isolate. Chloramphenicol, ofloxacin, Tobramycin, and Ciprofloxacin showed high sensitivity. Fusidic acid and Ceftazidime exhibited negligible sensitivity.

## Background

Cataract surgery, an intraocular surgery, is considered one of the most frequent procedures performed in the ophthalmology unit globally [1]. The number of patients who undergo this type of surgery has increased continuously amongst the Iraqi population over the past years [2, 3]. Thus, postoperative endophthalmitis represents a serious complication. Its prevalence rate has been estimated to be between 0.07% and 0.12% [4–6]. Endophthalmitis is an inflammatory condition of the eye often caused by bacterial infection. Most bacteria responsible for postoperative ocular infection are part of the normal microbial flora of the conjunctiva and eyelids of the patients [7, 8]. The appearance of this complication mostly leads to worsening of patients’ vision and may result in permanent loss of vision if not treated properly showcasing a poor prognosis [9]. Coagulase-negative staphylococcus, Staphylococcus aureus, and Streptococcus spp. are the common causes of postoperative endophthalmitis [10]. Gram-negative organisms are responsible for up to 15% of the infections [11] Multiple bacterial species prevail in a vaginally delivered infant’s eye, including Staphylococcus aureus, Staphylococcus epidermidis, Streptococci, and Escherichia coli; Streptococci and Pneumococci then predominate during the first two decades of life. The most prevalent species detected from the external eye are Staphylococcus epidermidis and other coagulase-negative Staphylococci (CoNS), Staphylococcus aureus, and diphtheroids [12]. 

The source of pathogens for endophthalmitis is mainly the ocular surface of the patients. Additionally, nasal mucosa serves as a reservoir of organisms for the conjunctiva and nasolacrimal system. Speaker et al. demonstrated that organisms isolated from the vitreous of endophthalmitis were genetically indistinguishable from those derived from the eyelids, conjunctiva and noses in 82% of cases [7]. Consequently, the authors suggested to provide special attention to the external tissues, and their microbial flora in the prophylaxis and prevention of postoperative bacterial endophthalmitis. Evidence shows that surface flora routinely gain entry to the anterior chamber during cataract surgery. In one study, 13 of 59 eyes (22%) grew gram-positive organisms from aqueous cultures taken at the time of wound closure after intraocular surgery, and 8 of these 13 eyes (62%) had organisms with identical species and antibiotic sensitivities to organisms isolated from the eyelids and conjunctiva before or after disinfection [13]. 

Post cataract endophthalmitis is a potentially devastating event for patients, and the efforts made to reduce its prevalence led to a progressive decrease over time, currently ranging from 0.014% in USA [14] and 0.027% in Sweden [15] to 0.031% in Spain [16]. Use of prophylactic antibiotics in cataract surgery reduces the number of organisms in the conjunctiva and eyelids and thus, reduces the risk of postoperative infection [17–19]. Trends of bacterial resistance have been shown to increase among commonly used antibiotics such as penicillin, erythromycin, and tetracycline [20] Resistance to antibiotics continues to emerge worldwide, with multidrug-resistant organisms become increasingly common. The mechanisms by which bacteria develop antibiotic resistance at the cellular level are mutations and genetic exchange. The extended use of antimicrobials not only in hospitals but also in long-term or day-care facilities, outpatient settings, industrial livestock production, and veterinary care, all promote the development and survival of resistant bacterial strains. in order to allow the best pre-, peri-, and post-operative management, additional studies are needed in order to gain a deeper knowledge of the ocular bacterial flora, and their antibiotic resistance [21]. Considering the surface microbial flora is one of the major causes of endophthalmitis, the aim of the present study was to investigate the pre-operative conjunctival flora and their antibiotic sensitivity in a group of subjects undergoing cataract surgery.

## Patients and methods

### Study design

A cross-sectional study was conducted on patients scheduled for cataract surgery. at Ibn Al-Haitham Teaching Eye Hospital, a tertiary ophthalmology center in Baghdad, Iraq.

### Study approval and ethical considerations

The required official approvals were obtained before the initiation of data collection. These included the approval from The Arab Board of Health Specializations and the scientific committee of Ibn Al-Haitham Teaching Eye Hospital. Informed consent was obtained from all participants after explaining the study’s objectives. Confidentiality was ensured by anonymizing patient information using unique identification codes.

### Study participants

A total of 200 patients (200 eyes) scheduled for cataract surgery (phacoemulsification or extracapsular extraction) between January and November 2023 were included. Conjunctival swabs were taken from a single eye if the patient had bilateral cataract. The sample size was calculated using the formula for estimating a single population proportion:

$$\:\varvec{N}\:=\frac{{\varvec{Z}}^{2\:}.\varvec{P}.(1-\varvec{P})}{{\varvec{E}}^{2\:}}\:$$

Where: Z = 1.96 (corresponding to a 95% confidence level), *P* =.5 (anticipated proportion of normal conjunctival flora carriage, chosen to maximize variability due to limited prior data in our setting), E = 0.07 (7% margin of error).

Patients were recruited during their biometry sessions, where they were informed about the study and invited to participate. Demographic data, including age, gender, and systemic comorbidities (e.g., diabetes, cardiovascular disease), were collected using a structured questionnaire.

All participants underwent comprehensive ophthalmic evaluations, including medical history and a thorough external ocular examination using a slit-lamp biomicroscope to confirm the absence of active ocular infections or inflammation.

The inclusion criteria were patients admitted for cataract surgery. Patients were excluded if they had current or recent (within one month) use of topical or systemic antibiotics, or if they were using any topical eye medications, including steroids. Exclusion also applied to those with systemic immunosuppression, use of immunosuppressive drugs, or a history of uveitis, glaucoma, previous ocular surgery, or trauma. Patients with a history of contact lens use, congenital cataracts, or active ocular surface diseases (such as anterior blepharitis, meibomitis, dry eye, chronic dacryocystitis, or conjunctivitis) were also excluded.

### Data collection

Conjunctival swabs were collected in a sterile surgical environment on the day of surgery before the application of topical anesthetics, antibiotics, or povidone-iodine. Samples were obtained from the inferior conjunctival sac using sterile cotton-tipped applicators, avoiding contact with the cornea, lid margins, or eyelashes. Swabs were immediately placed in sterile transport media and delivered to the microbiology laboratory within one hour.

### Laboratory procedures

#### Bacterial isolation and identification

- Sample Inoculation: Conjunctival swabs were aseptically inoculated onto blood agar, chocolate agar, and MacConkey agar plates using the streaking method. Plates were incubated aerobically at 37 °C for 24–48 h.
- Gram Staining: Isolates were categorized as Gram-positive or Gram-negative based on cell wall morphology. Gram-positive bacteria were identified as cocci (spherical cells) under microscopy, with Staphylococcus species forming grape-like clusters and Streptococcus appearing in chains or pairs.

Biochemical Differentiation:

- Gram-Positive Bacteria:

Catalase Test: Used to differentiate Staphylococcus (catalase-positive) from Streptococcus (catalase-negative).

Coagulase Test: Staphylococcus aureus (coagulase-positive) was distinguished from coagulase-negative staphylococci (e.g., Staphylococcus epidermidis).

Mannitol Salt Agar (MSA): Staphylococcus aureus fermented mannitol (yellow colonies), while other staphylococci did not.

Additional Tests: For Streptococcus species, bile esculin hydrolysis and optochin susceptibility tests were performed to differentiate Streptococcus pneumoniae from viridans group streptococci. Ambiguous isolates underwent API Staph biochemical profiling (BioMérieux, France).

- Gram-Negative Bacteria:

Lactose Fermentation: MacConkey agar identified lactose-fermenting colonies (e.g., E coli: pink colonies).

Biochemical Confirmation:

IMViC Tests: E. coli was confirmed by indole production (+), methyl red (+), Voges-Proskauer (–), and citrate (–).

Triple Sugar Iron (TSI) Agar: Acidic butt/slant with gas production (A/A + gas).

Eosin Methylene Blue (EMB) Agar: Metallic green sheen indicated E. coli.

#### Fungal isolation and identification

Culture: Suspected fungal isolates were subcultured on Sabouraud Dextrose Agar (SDA) and incubated at 25–30 °C for 3–7 days.

Microscopy: Lactophenol cotton blue staining revealed septate hyphae with conidial structures (e.g., *Aspergillus* species displaying characteristic radiating conidial heads).

#### Quality control

Reference strains (Staphylococcus aureus; ATCC 25923, E coli; ATCC 25922) were included for biochemical test validation.

### Antibiotic susceptibility testing

Antibiotic susceptibility was determined using the disc diffusion method on Mueller-Hinton agar following CLSI guidelines [22]. The antibiotics tested included ciprofloxacin, moxifloxacin, ofloxacin, gentamicin, tobramycin, ceftazidime, cefuroxime, vancomycin, fusidic acid, and chloramphenicol. For each bacterial isolate, two Mueller-Hinton agar plates were prepared, with five antibiotic discs placed on each plate. Plates were incubated at 35 °C overnight, and inhibition zones were measured the next day. Results were interpreted using an antimicrobial susceptibility chart.

### Data analysis

Data were processed and analyzed using IBM SPSS Statistics Version 23. Descriptive statistics were applied, with categorical data presented as frequencies and percentages and continuous variables as means with standard deviations. The chi-square test was used for categorical variables, while independent samples t-tests were used to compare age between groups. A *p*-value of less than 0.05 was considered statistically significant.

## Results

A total of 200 patients (200 eyes) scheduled for cataract surgery (phacoemulsification or extracapsular extraction) between January and November 2023 were included, with a mean age of 60.79 ± 9.93 years. The majority of the participants were male 131 (65.5%), while females constituted 69 (34.5%). Among the eyes studied, 108 (54%) were right eyes, and 92 (46%) were left eyes. Of the participants, 21 (10.5%) were prepared for Extracapsular cataract extraction (ECCE), while 179 (89.5%) were scheduled for phacoemulsification. *(*Table 1*)*

**Table 1:** Baseline characteristics of included participants

Variables	Negative cultures	Positive cultures	Total	*P*-value
Age in years (Mean ± SD)	60.03 ± 9.6	63.19 ± 10.69	-	0.190*
Gender: Male	98 (74.8%)	33 (25.2%)	131 (65.5%)	0.587**
Gender: Female	54 (78.3%)	15 (21.7%)	69 (34.5%)	
Laterality: Right	83 (76.9%)	25 (23.1%)	108 (54%)	0.760**
Laterality: Left	69 (75%)	23 (25%)	92 (46%)	
Smoking: No	97 (76.4%)	30 (23.6%)	127 (100%)	0.869*
Smoking: Yes	55 (75.3%)	18 (24.7%)	73 (100%)	
Diabetes mellitus: No	102 (74.5%)	35 (25.5%)	137 (100%)	0.450*
Diabetes mellitus: Yes	50 (79.4%)	13 (20.6%)	63 (100%)	

*: Independent Samples T-test

**: Chi-square test

Culture results revealed that 152 (76%) were negative, and 48 (24%) were positive. The distribution of isolates from positive cultures included *S. epidermidis* 36 (75%), *S. aureus* 7 (14.58%), *Aspergillus species* 3 (6.25%), and *E. coli* 2 (4.17%). *(*Table 2*)*There was no statistically significant difference in mean age between participants with positive and negative cultures, with a minimal difference of only 3.16 ± 14.3. Similarly, bacterial growth showed no association with gender, as 74.8% of males and 78.3% of females had negative cultures. Smoking status also did not influence culture results, with positive cultures found in 23.6% of non-smokers and 24.7% of smokers (*P* =.869). Likewise, diabetes mellitus was not significantly associated with bacterial growth, with 25.5% of non-diabetics and 20.6% of diabetics showing positive cultures (*P* =.450). Laterality also was not related to growth results (*P =.760*). *(*Table 1*)*

**Table 2:** Microbial isolates and antibiotics sensitivity status of included participants

Microbe isolate	S. Epidermidis (*N* = 36)			S.aureus (*N* = 7)			E.coli (*N* = 2)		Total (*N* = 45)	
Antibiotics sensitivity	Sensitive	Intermediate	Resistant	Sensitive	Intermediate	Resistant	Sensitive	Resistant	Sensitive	Resistant
Ofloxacin	35 (97.2%)	-	1 (2.8%)	4 (57.1%)	-	1 (14.2%)	2 (100%)	-	41 (91.1%)	2 (4.4%)
Chloramphenicol	34 (94.4%)	-	2 (5.6%)	7 (100%)	-	-	2 (100%)	-	43 (95.6%)	2 (4.4%)
Ciprofloxacin	32 (88.9%)	1 (2.8%)	3 (8.3%)	3 (42.8%)	-	2 (28.5%)	2 (100%)	-	37 (82.2%)	3 (6.7%)
Tobramycin	30 (83.3%)	1 (2.8%)	6 (16.7%)	6 (85.7%)	-	1 (14.2%)	2 (100%)	-	38 (84.4%)	2 (4.4%)
Gentamycin	25 (69.4%)	-	11 (30.6%)	3 (42.8%)	1 (14.2%)	4 (57.1%)	2 (100%)	-	30 (66.7%)	15 (33.3%)
Moxifloxacin	24 (66.7%)	2 (5.6%)	10 (27.8%)	3 (42.8%)	2 (28.5%)	2 (28.5%)	2 (100%)	-	29 (64.4%)	4 (8.9%)
Vancomycin	22 (61.1%)	-	14 (38.9%)	5 (71.4%)	2 (28.5%)	2 (28.5%)	1 (50%)	1 (50%)	28 (62.2%)	17 (37.8%)
Cefuroxime	21 (58.3%)	1 (2.8%)	14 (38.9%)	5 (71.4%)	-	2 (28.5%)	2 (100%)	-	28 (62.2%)	16 (35.6%)
Fusidic Acid	4 (11.1%)	-	32 (88.9%)	-	-	-	2 (100%)	-	6 (13.3%)	32 (71.1%)
Ceftazidime	2 (5.6%)	-	34 (94.4%)	-	-	-	2 (100%)	-	4 (8.9%)	34 (75.6%)

The highest antibiotic sensitivity among the bacterial isolates was observed with chloramphenicol (95.6%), followed by ofloxacin (91.1%), tobramycin (84.4%), and ciprofloxacin (82.2%). Lower sensitivities were recorded with moxifloxacin (64.4%) and gentamicin, cefuroxime, and vancomycin (each at 62.2%). Notably, fusidic acid demonstrated a sensitivity of only 8.9%, and ceftazidime had the lowest sensitivity at 4.4%. *(*Table 2*)*

For *S. epidermidis*, the highest sensitivities were with ofloxacin (97.2%), chloramphenicol (94.4%), ciprofloxacin (88.9%), and tobramycin (83.3%). Moderate sensitivities were observed with gentamicin (69.4%), moxifloxacin (66.7%), vancomycin (61.1%), and cefuroxime (58.3%). The lowest sensitivities were with fusidic acid (11.1%) and ceftazidime (5.6%). *(*Table 2*)*

In the case of *S. aureus*, chloramphenicol exhibited the highest sensitivity (100%), followed by tobramycin (85.7%) and cefuroxime and vancomycin (each at 71.4%). Ofloxacin showed moderate sensitivity (57.1%), while ciprofloxacin, moxifloxacin, and gentamicin each had sensitivities of 42.8%. Fusidic acid and ceftazidime exhibited no sensitivity (100% resistance). The two *E. coli* isolates demonstrated 100% sensitivity to ciprofloxacin, ofloxacin, moxifloxacin, tobramycin, cefuroxime, and chloramphenicol. Vancomycin exhibited 50% sensitivity, while gentamicin, fusidic acid, and ceftazidime showed no sensitivity (100% resistance). *(*Table 2*)*

Regarding antibiotic resistance, 30.6% of *S. epidermidis* isolates were resistant to two antibiotics, while 16.7% exhibited resistance to four and six antibiotics, respectively. A single isolate (2.8%) demonstrated resistance to seven antibiotics. Among *S. aureus* isolates, 28.6% were resistant to three and six antibiotics, respectively. The two *E. coli* isolates displayed resistance to three and four antibiotics, respectively. *(*Table 2*)*

## Discussion

Although substantial evidence exists regarding the efficacy and safety of intracameral antibiotics, topical antibiotics are still the predominant form of prophylaxis for postoperative endophthalmitis employed by surgeons [23]. Understanding the spectrum of the ocular flora and their antibiotic susceptibility in various geographic locales can assist clinicians in optimizing prophylactic antibiotic treatments. We herein present results from a cross-sectional of patients who were scheduled to undergo cataract surgery at our tertiary eye hospital. It is well known that other bacteria such as Propionibacterium and Corynebacterium spp may be found on the ocular surfaces [24], but none of these were isolated in the present study. S.epidermidis, S.aureus and other (which are usually found as normal flora) have been implicated as potential causes of post-surgical endophthalmitis [7, 8, 10], thus their identification from preoperative patients in this study calls for introduction an ongoing surveillance to establish the trend essential for infection control in this setting. Furthermore, the incidence rate of postoperative endophthalmitis following cataract surgery has surprisingly increased, 3,140,650 patients who underwent cataract surgeries in the period 1963–2003, the incidence of an infection in the period 1963–2003 was 0.128%, while it accounted for 0.265% in the period from 2000 to 2003 [25]. Moreover, Recent datasets have reported incidence rates for post-cataract endophthalmitis ranging from 0.1 to 0.5 per 1000 cases [26, 27]. There are a number of risk factors related to postoperative infection, including age, underlying disease, history of use of antibiotics and steroids, types of cataract surgery, intraoperative complications, postoperative antibiotic use, and patients’ personal hygiene [25–27]. 

Antibiotic use is now prevalent, and sometimes, it is overused. Overuse of antibiotics can result in an increase of drug resistance or, even worse, alteration of normal flora. Therefore, this study aimed to study the normal flora of the eye and its sensitivity to the commonly used antibiotics, the exclusion of specific patient groups in this study was necessary to minimize confounding factors that could influence the results. Patients currently using topical or systemic antibiotics, or with a history of their use within one month prior to sample collection, were excluded to avoid skewing the bacterial culture results, as recent antibiotic exposure could suppress bacterial growth and alter antibiotic sensitivity patterns [28]. Similarly, patients using other topical eye drops, including steroids, were excluded because these could also affect the ocular surface’s microbiota by reducing inflammation or altering the local environment [29]. Individuals with systemic immunosuppression or those on immunosuppressants were excluded to eliminate the influence of compromised immunity, which could increase susceptibility to infections or alter the conjunctival flora [30]. Patients with a history of uveitis, glaucoma, previous ocular surgery, or ocular trauma were excluded as these conditions could introduce structural or microbiological changes to the ocular surface, potentially influencing culture results [31–34]. Furthermore, patients with a history of contact lens wear were excluded due to the increased risk of microbial contamination associated with contact lens use [35]. By excluding these patients, the study aimed to focus on a relatively homogenous group undergoing cataract surgery to ensure the accuracy and reliability of the findings regarding conjunctival flora and antibiotic resistance patterns.

The current study found no statistically significant association between bacterial growth and age, gender, laterality, smoking, or diabetes mellitus. This may be due to the stringent exclusion criteria, which minimized variations in conjunctival flora by focusing on a relatively healthy population. Age-related immune differences or gender-based hormonal effects were likely negligible, while the uniform environmental exposure of both eyes explains the lack of association with laterality. Smoking and diabetes may have limited impact on conjunctival flora in the absence of systemic or ocular complications. These findings highlight the need for further studies in larger, more diverse populations to explore these relationships.

In this study, 152 (76%) cultures were negative, while 48 (24%) showed positive growth. The most common organism isolated was coagulase-negative *Staphylococcus epidermidis* (75%), followed by *S. aureus* (14.58%), *Aspergillus spp.* (6.25%), and *E. coli* (4.17%). The positive growth rate in this study was quite lower compared to other studies from Brazil (86%) [11], United States (85.2%) [36], India (71%) [37], Philippines (61.6%) [38], India (58%) [39], Iran (52.4%) [40], Oman (48.3%) [41], Nigeria (46.3%) [42], Uganda (45.8%) [43]. Results from Thailand (30.8%) [44], Taiwan (26.6%) [45], and Italy (25.3%) [46] were nearly comparable to ours. Conversely, AboElnour A et al. [47] study from Egypt reported only (9%) positive culture. (Table 3) Differences in culture yield could be due to insufficient rotation of the swab on the conjunctiva, lengthy shipping times, or differences in culture conditions. Additionally, the prevalence of antibiotic use may vary across regions, contributing to differences in susceptibility patterns. Additionally, as observed in Satish K et al. [37], patients with sterile conjunctiva often report prior use of antibiotics, which may suppress growth, the low growth percentage compared to other studies may be attributed to the widespread misuse or abuse of antibiotics (topical or systemic) despite excluding patients with a history of antibiotic use in the previous month. In our study, *E. coli* was detected in conjunctival swabs, a finding not reported in similar research. This unexpected result may reflect environmental factors unique to Baghdad, variations in healthcare practices, or regional differences in conjunctival microbiota. Furthermore, patient-related hygiene practices and undiagnosed systemic conditions may have contributed to this observation. These findings highlight the need to consider geographic and environmental influences when assessing conjunctival flora.

**Table 3:** Comparison between the current study and similar studies regarding the percentage of positive cultures and percentage of cons from all isolates

Study	Location	Year	No. of swabs	Positive cultures percentage	CoNS * percentage
The current study	Iraq	2020	200	24%	75%
AboElnour et al. [47]	Egypt	2015	11,468	9.0%	57%
Bruttini C et al. [46]	Italy	2020	83	25.3%	15%
Lin et al. [45]	Taiwan	2017	128	26.6%	45.2%
Ratnumnoi et al. [44]	Thailand	2017	120	30.8%	97.2%
Mshangila et al. [43]	Uganda	2013	131	45.8%	65%
Iorwuese et al. [42]	Nigeria	2018	134	46.3%	74.8%
Keshav et al. [41]	Oman	2012	112	48.3%	81.4%
Ansari et al. [40]	Iran	2008	170	52.4%	88.7%
Karthika et al. [39]	India	2014	100	58%	55.1%
Cham et al. [38]	Philippines	2009	60	61.6%	89%
Satish et al. [37]	India	2019	58	71%	39%
Hsu et al. [36]	USA	2013	183	85.2%	74.8%
Arantes et al. [11]	Brazil	2006	50	86%	54%

* CoNS: coagulase-positive staphylococci

Notably, only two studies were previously conducted from Iraq during 2007–2009 regarding the conjuctival flora, Kareem et al. [48] reported 39% positive conjunctival cultures while El-Nahi et al. [49] revealed 51% positive growth pre- and one day post-cataract extraction. The variability of results could be subjected to unclear inclusion/exclusion criteria of both studies, as Kareem et al. [48] didn’t state any criteria while only brief exclusion criteria were described by El-Nahi et al. Similarly, both studies yielded S.epidermidis as the most prevalent isolate [48, 49]. 

The antibiotic sensitivity profile of all bacterial isolates showed high sensitivity to chloramphenicol (95.6%), ofloxacin (91.1%), tobramycin (84.4%), and ciprofloxacin (82.2%), while the lowest sensitivities were seen with fusidic acid (8.9%) and ceftazidime (4.4%). Isolates of S.epidermidis showed the highest sensitivities to ofloxacin (97.2%) and chloramphenicol (94.4%) but the lowest sensitivities to ceftazidime (5.6%) and fusidic acid (11.1%). These results may reflect the widespread use of specific antibiotics in Iraq. For instance, indiscriminate use and self-medication with fusidic acid may account for its low sensitivity. While chloramphenicol is less frequently prescribed due to its potential side effects, which may explain its high sensitivity in this study. Regarding chloramphenicol, our results were consistent with previous studies by Arantes et al. (92%) [11], Cham et al. (89%) [38], lorwuese et al. (96.9%) [42], AboElnour et al. (88.5%) [47],. However, the lowest chloramphenicol sensitivity was observed in Keshav et al. Study (63%) [41]. (Table 4). Further, ofloxacin high sensitivity in our study goes in prallel with reports by Arantes et al. (97.4%) [11], Cham et al. (92%) [38], AboElnour et al. (74.3%) [47]. Ciprofloxacin showed high sensitivity within majority of previous research [11, 40–43, 45, 47] apart from Hsu et al. [36] where ciprofloxacin deemed to be less sensitive (64%). This could be explained by the inclusion of all positive cultures only in the study of Hsu et al. [36].

**Table 4:** Comparison between the current study and similar studies regarding the antibiotic sensitivity profile

Studies		Chloramphenicol	Ofloxacin	Tobramycin	Ciprofloxacin	Moxifloxacin	Gentamycin	Vancomycin	Cefuroxime	Fusidic Acid	Ceftazidime
The current study	All isolates	95.6%	91.1%	84.4%	82.2%	64.4%	64.4%	64.4%	62.2%	8.9%	4.4%
	*S. Epidermidis* isolates	94.4%	97.2%	83.3%	88.9%	66.7%	69.4%	61.1%	58.3%	11.1%	5.6%
AboElnour et al. [47]	All isolates	88.5%	74.3%	85.6%	71.8%	-	82.4%	87.7%	-	42.7%	-
	CoNS ^*^ isolates	84.3%	74.3%	87%	75%	-	74.2%	86%	-	44.2%	-
Lin et al. [45] (All staphylococcal isolates)		-	-	55%	80%	82.5%	-	100%	-	-	-
Ratnumnoi et al. [44] (All isolates)		-	-	98.67%	-	100%	-	-	-	-	-
Iorwuese et al. [42] (All isolates)		96.9%	-	-	100%	-	100%	-	-	-	-
Mshangila et al. [43]	CoNS isolates	70.3%	-	74.7%	75%	-	79.1%	100%	-		-
	*S. Aureus* isolates	72.4%	-	82.8%	72.4%	-	69%	100%	-		-
Keshav et al. [41]	All isolates	63%	-	-	72%	-	74%	83%	6%	26%	-
	CoNS isolates	68%	-	-	84%	-	87%	98%	-	30%	-
Ansari et al. [40]	All isolates	-	-	-	100%	-	89.5%	58.2%	-	-	45.6%
	CoNS isolates	-	-	-	100%	-	89.9%	55.8%	-	-	47.7%
Cham et al. [38]	All isolates	89%	92%	79%	82%	92%	85%	93%	-	-	-
	CoNS isolates	86%	94%	85%	91%	95%	87%	100%	-	-	-
Satish et al. [37]	CoPS ^*^ isolates	-	-	-	80%	-	-	100%	-	-	-
	CoNS isolates	-	-	-	80%	-	-	80%	-	-	-
Hsu et al. [36]	All G + ve isolates	-	-	-	64%	75%	95%	100%	-	-	-
	*S. Epidermidis* isolates	-	-	-	63%	75%	94%	100%	-	-	-
Arantes et al. [11]	All isolates	92.1%	97.4%	-	89.5%	-	79%	89.5%	-	-	-
	CoNS isolates	92.6%	96.3%	-	88.9%	-	85.2%	100%	-	-	-

* CoNS: coagulase-positive staphylococci

** CoPS: coagulase-negative staphylococci

A notable finding in this study was of multidrug-resistant (MDR) isolates. Of the bacterial isolates, 93.3% were resistant to ≥ 2 antibiotics, and 66.6% were resistant to ≥ 3 antibiotics, which is significantly higher than rates reported in other studies. For example, AboElnour et al. [47] reported 28.2% resistance to ≥ 3 antibiotics, while Ansari et al. [40] observed 8.2% resistance in S. epidermidis. This growing trend in MDR bacteria highlights the urgent need for ongoing antimicrobial resistance surveillance.

This study’s strengths include; First, our findings directly inform ophthalmologists and microbiologists about common pathogens and their resistance profiles in conjunctival infections, aiding in empirical therapy decisions in the Iraqi population. Second, establishes a reference for monitoring emerging resistance or shifts in conjunctival flora over time. Third, cross-sectional design has minimized selection bias through consecutive enrollment of eligible participants. Fourth, adherence to institutional review board protocols ensured patient rights and data integrity.

Although, the study has several drawbacks. First, while the sample size is suitable for detecting typical microbial patterns, it may not capture rare or low-prevlance organisms. Second, the study’s single-center design limits the findings’ generlizability to other locations with differing demographics and exposures. Third, reliance on phenotypic methods may underdetect fastidious or genetically diverse species. Moreover, fungal isolates (e.g. Aspargillus) were identified morphologically but were not subjected anifungal susceptibility testing, limiting insights into resistance patterns. Finally, while the exclusion of patients on topical or systemic antibiotics reduced confounding, it may restrict the findings’ relevance to larger patient populations.

## Conclusion

This study found conjunctival microbial colonization in 24% of cataract surgery candidates, with coagulase-negative S. epidermidis being the most predominant isolate. Chloramphenicol, ofloxacin, Tobramycin, and Ciprofloxacin respectively demonstrated high sensitivity suggesting potential value in prophylactic regimens. Fusidic acid and Ceftazidime exhibited negligible sensitivity.

## Data Availability

No datasets were generated or analysed during the current study.
